# Changing Epidemiological Pattern and Higher Disease Burden of Influenza in China, 2022 to 2025

**DOI:** 10.1111/irv.70151

**Published:** 2025-08-30

**Authors:** Hairu Yu, Qing Wang, Boer Qi, Jie Qian, Weizhong Yang, Luzhao Feng

**Affiliations:** ^1^ School of Population Medicine and Public Health, Chinese Academy of Medical Sciences and Peking Union Medical College Public Health Emergency Management Innovation Center, State Key Laboratory of Respiratory Health and Multimorbidity, Key Laboratory of Pathogen Infection Prevention and Control (Peking Union Medical College), Ministry of Education Beijing China

**Keywords:** disease burden, epidemiological pattern, influenza

## Abstract

Influenza activity peaks in southern (59.62%) and northern China (57.60%) during the 2022/2023 season reached the highest levels in the past 10 years. The 2023/2024 season witnessed a longer duration of the winter–spring epidemic weeks and a higher disease burden compared with previous high‐epidemic years. The A(H3N2), A(H1N1)pdm09, and B/Victoria lineages alternated among the predominant circulating strains from the 2022/2023 season to the 2024/2025 season. After the 2022/2023 and 2023/2024 seasons of disruption and fluctuation, influenza in the 2024/2025 season has reverted to its previous epidemic pattern.

## 1 Introduction

Accumulated evidence [1] indicates the influenza‐associated all‐cause mortality rate in China is 14.33 per 100,000 persons during 2005–2019. Based on surveillance data in China from 2022 to 2025, this study analyzed the trends in epidemiology, virological characteristics, and disease burden to provide evidence for improving influenza protection and control strategies.

## 2 Methods

### 2.1 Data Sources

Based on the Influenza Weekly Report of the China Center for Disease Control and Prevention, we collected data on the test‐positive rate, the percentage of influenza‐like illness among total outpatient and emergency department visits (ILI%), and virological surveillance of the southern and northern China from Week 14 of 2022 to Week 13 of 2025.

### 2.2 Relevant Definitions

1. Influenza activity intensity [2]: The average positive rate among all epidemic weeks of one surveillance year was classified into high(≥ 25%), moderate(≥ 20% to <25%), and low(20%) levels.
2. Influenza attack rate:
$$\begin{array}{c} \begin{array}{c} \frac{\text{The total population of research region}\times \text{outpatient rate}\times \mathrm{ILI}\%\times \text{test}-\text{positive rate of influenza}}{\text{The total population of research region}\times \left(1-\text{influenza vaccination coverage}\times \text{vaccine protective rate}\right)} \end{array} \end{array}$$

The attack rate was defined as “The proportion of an exposed population that develops influenza within a specified period [3].” In this study, the attack rate was used as the indicator of disease burden. We indirectly estimated the influenza attack rate by leveraging real‐world vaccination coverage and outpatient rate, applying a self‐comparison method across different surveillance years in southern and northern China. We simplified the equation by cancellation of constants and addressed the challenge of limited access to certain real‐time parameters. Specifically, since 2022, the total population, the outpatient rate, and the influenza vaccine efficacy remained relatively stable, and influenza vaccination coverage in mainland China has remained low without abrupt increases. As such, we used the weekly product of ILI% and influenza test‐positive rate as a composite parameter to construct a time‐series curve. The area under the curve (AUC) and its fold changes between influenza seasons were used as indirect measures of variation in confirmed influenza attack rate across different periods.

### 2.3 Statistical Analysis

R version 4.5.0 and Excel were used for descriptive analysis and Chi‐square test of influenza relevant data.

## 3 Results

### 3.1 Influenza Activity Intensity

The average positive rates in the south from the 2022/2023 to 2024/2025 seasons reached high epidemic levels (30.66%, 31.47%, and 25.45%, respectively). Those in the north reached high epidemic levels (26.73% and 26.95%) in the 2022/2023 and 2023/2024 seasons, while they decreased to a moderate epidemic level (21.88%) during the 2024/2025 season. Additionally, the activity peaks of the south (59.62%) and north (57.60%) in the 2022/2023 season have reached the highest levels in the past 10 years (*p* < 0.001) (Table 1).

**TABLE 1:** Influenza activity intensity and peak in China, 2013/2014 to 2024/2025 seasons.

Surveillance year	The south				The north			
	Activity intensity (%)	Peak (%)	χ^2^	*P*‐value	Activity intensity (%)	Peak (%)	χ^2^	*P*‐value
2013–2014	23.73	42.57^a^	2302.6	< 0.001	21.38	36.25^a^	1240.2	< 0.001
2014–2015	18.12	25.57^a^			18.66	39.12^a^		
2015–2016	21.10	33.78^a^			28.93	41.98^a^		
2016–2017	15.74	24.82^a^			20.03	26.73^a^		
2017–2018	26.65	47.97^a^			32.38	48.47^a^		
2018–2019	33.23	44.86^a^			31.87	41.00^a^		
2022–2023	30.66	59.62^#^			26.73	57.60^#^		
2023–2024	31.47	55.23^a^			26.95	43.93^a^		
2024–2025	25.45	35.75^a^			21.88	35.35^a^		

*Note:* Chi‐square test was used for overall testing of influenza activity peaks during each surveillance year, with Bonferroni correction applied to control the family‐wise error rate in multiple comparisons.

^#^ Reference group.

^a^ A statistically significant difference was shown when comparing to the reference group.

### 3.2 Seasonality and Virological Characteristics

**FIGURE 1:**
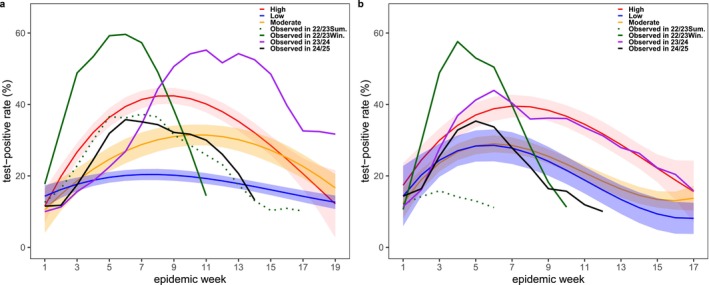
Observed influenza activity in 2022–2025 and fitted levels using 2013–2019 historical data. a, the south; b, the north.

China has a winter–spring epidemic peak each year (Figure 1). Notably, the high activity intensity in the south previously during summer disappeared in the 2023/2024 and 2024/2025 seasons. The duration of the winter–spring epidemic weeks in the south (28 weeks) and north (20 weeks) in the 2023/2024 season reached the highest levels in the past 5 years. The peak onset time in the south (Week 13 of 2023) and north (Week 10 of 2023) during the 2023/2024 season exhibited an approximately 10‐week delay in comparison with those from the 2016/2017 to 2018/2019 seasons (Table 2).

**TABLE 2:** Duration of the winter–spring epidemic weeks and peak onset time of influenza in China, 2013/2014 to 2024/2025 seasons.

Surveillance year	The south		The north	
	Duration of epidemic weeks	Peak onset time (nature week)	Duration of epidemic weeks	Peak onset time (nature week)
2013–2014	20	Week 2 of 2014	17	Week 14 of 2014
2014–2015	15	Week 4 of 2015	12	Week 52 of 2014
2015–2016	21	Week 12 of 2016	17	Week 11 of 2016
2016–2017	30	Week 51 of 2016	18	Week 52 of 2016
2017–2018	18	Week 3 of 2018	16	Week 2 of 2018
2018–2019	15	Week 4 of 2019	15	Week 3 of 2019
2022–2023	11	Week 13 of 2023	10	Week 10 of 2023
2023–2024	28	Week 50 of 2023	20	Week 49 of 2023
2024–2025	14	Week 2 of 2025	12	Week 1 of 2025

A(H3N2) was the predominant circulating strain through Week 6 of 2023. Subsequently, A(H3N2) and A(H1N1)pdm09 showed cocirculation characteristics. The A(H3N2) and B/Victoria lineages alternated with the predominant circulating strains during the 2023/2024 season. A(H1N1)pdm09 became the predominant circulating strain during the 2024/2025 season (Figures S1 and S2).

### 3.3 Disease Burden

**FIGURE 2:**
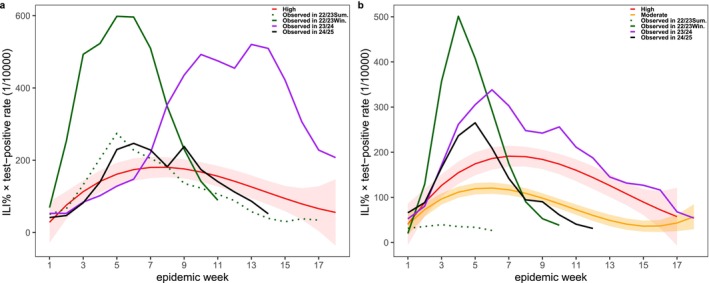
Observed influenza disease burden in 2022–2025 and fitted levels using 2013–2019 historical data. a, the south; b, the north.

The disease burden in the south during the 2022/2023 season reached 1.88 times the previous high epidemic level, while that in the north was 0.84 times the previous high. The disease burden in the south during the 2023/2024 season reached 2.12 times the previous high epidemic level, while that in the north was 1.65 times the previous high. The disease burden in the south during the 2024/2025 season was 0.64 times the previous high epidemic level, while that in the north was 1.11 times the previous moderate epidemic level (Figure 2).

## 4 Discussion

Implementing systematic surveillance and summarizing the dynamics of influenza are crucial for prevention and control. This study found that the 2022/2023 season witnessed the highest activity peak in the last 10 years. Other studies have reported this phenomenon [4, 5]. First, due to increasing population, more frequent indoor activity, and climate change, the risk of influenza transmission has intensified. Second, disparities in immune protection levels among different populations resulting from different prior immune exposure histories have aggravated the intensity of influenza activity in China [6, 7]. A summer epidemic level occurred in northern China, and there was no influenza peak in winter during the 2022/2023 season. Instead, there was a delayed epidemic peak in spring, which may be related to the late 2022 epidemic of SARS‐CoV‐2 in China, leading to a delayed peak in influenza and a “see‐saw” effect [8].

Accumulated evidence [1, 9, 10] indicates influenza epidemics contributed to a severe disease burden. This study revealed that the 2023/2024 season had the highest disease burden in the last decade, which was consistent with the findings of Du [11, 12]. First, the activity intensity in the 2023/2024 season reached a high epidemic level. Second, the duration of the winter–spring epidemic weeks in the southern and northern China during the 2023/2024 season were 28 and 20 weeks, respectively. The combination of high activity intensity and protracted duration of epidemic weeks in the 2023/2024 season contributed to a severe disease burden. Zhao's study found that influenza mortality rates increased by 366.67% and showed a significantly increased trend during 2004–2020 [13]. A study from Zhejiang province in China also comprehensively considered the influenza test‐positive rate as well as ILI% to estimate the incidence, indicating a monotonically increasing trend [14].

In summary, during the atypical variations of the 2022/2023 and 2023/2024 seasons, the epidemiological characteristics of influenza showed a normal trend. This suggests the prevention and control of influenza could return to the regular standard, and strengthening influenza vaccinations remains an effective measure to protect susceptible populations [15].

## 5 Conclusion

The 2022/2023 and 2023/2024 influenza seasons were characterized by disruption and fluctuation. Influenza exhibited high epidemic levels and several influenza virus subtypes, except for the B/Yamagata lineage, which alternated with the predominant circulating strain from the 2022/2023 to 2024/2025 seasons. Since the 2024/2025 season, influenza has been reverting to its previous epidemic pattern.

## Author Contributions

**Hairu Yu:** conceptualization, software, data curation, formal analysis, writing – original draft, writing – review and editing, visualization. **Qing Wang:** conceptualization, software, writing – review and editing, methodology, supervision, validation. **Boer Qi:** writing – review and editing. **Jie Qian:** funding acquisition, writing – review and editing. **Weizhong Yang:** writing – review and editing, funding acquisition, supervision. **Luzhao Feng:** conceptualization, methodology, funding acquisition, supervision, writing – review and editing, project administration, resources.

## Ethics Statement

The authors have nothing to report.

## Conflicts of Interest

The authors declare no conflicts of interest.

## Peer Review

The peer review history for this article is available at https://www.webofscience.com/api/gateway/wos/peer‐review/10.1111/irv.70151.

## Supporting information


**Figure S1:** Observed virological characteristic of influenza in the south during 2022–2025.
**Figure S2:** Observed virological characteristic of influenza in the north during 2022–2025.

## Data Availability

The data that support the findings of this study are openly available in the Influenza Weekly Report of China Center for Disease Control and Prevention at https://ivdc.chinacdc.cn/cnic/.
