# Supplementary material for: Changing Epidemiological Pattern and Higher Disease Burden of Influenza in China, 2022 to 2025
Source: Influenza Other Respir Viruses. 2025 Aug 30;19(9):e70151. doi: 10.1111/irv.70151 (PMC12397873; doi:10.1111/irv.70151)
Supplement: Supplementary file 1 — Figure S1: Observed virological characteristic of influenza in the south during 2022–2025. Figure S2: Observed virological characteristic of influenza in the north during 2022–2025. [file IRV-19-e70151-s001.docx]

**Relevant definitions**

1. Influenza surveillance year: A surveillance year was defined as the period from the week 14 of each year to week 13 of next year .
2. Quarter: Spring was defined as the period from the week 10 to the week 22 annually. Summer was defined as the period from the week 23 to the week 35 annually. Autumn was defined as the period from the week 36 to the week48 annually. Winter was defined as the period from the week 49 to the week 9 of the next year.
3. Influenza season: The start point was defined as the first week of the test-positive rates greater than 10% for more than consecutive weeks and the ending point was the last week of the test-positive rates greater than 10% for more than consecutive weeks. The period from the start point to the ending point was defined as influenza season.
4. Activity intensity: The test-positive rates greater than 10% for more than consecutive weeks were considered as epidemic week.

**Influenza activity intensity**

Based on the average test-positive rate among all epidemic weeks of one surveillance year, the high epidemic years in the South were the 2017/18 season and 2018/19 season, respectively. The moderate epidemic years in the South were the 2013/14 season and 2015/16 season, respectively. The low epidemic years in the South were the 2014/15 season and 2016/17 season, respectively.The high epidemic years in the North were the 2015/16 season, 2017/18 season and 2018/19 season, respectively. The moderate epidemic years in the North were the 2013/14 season and 2016/17 season, respectively. The low epidemic years in the North was the 2014/15 season.

**Seasonality and virological characteristics**

**Figure S1 Observed virological characteristic of influenza in the South during 2022-2025**

**Figure S2 Observed virological characteristic of influenza in the North during 2022-2025**
